# A month in review: longitudinal dynamics between daily PTSD symptom networks, affect, and drinking behaviors in female college students

**DOI:** 10.3389/fpsyg.2024.1388539

**Published:** 2024-07-30

**Authors:** Stephanie Balters, Marc Schlichting, Thomas O. Walton, Mykel J. Kochenderfer, Debra Kaysen

**Affiliations:** ^1^Department of Psychiatry and Behavioral Sciences, School of Medicine, Stanford University, Stanford, CA, United States; ^2^Department of Aeronautics and Astronautics, School of Engineering, Stanford University, Stanford, CA, United States; ^3^Department of Psychiatry and Behavioral Sciences, School of Medicine, University of Washington, Seattle, WA, United States

**Keywords:** posttraumatic stress disorder (PTSD), affect, self-medication, alcohol abuse, women’s health, symptom networks

## Abstract

**Introduction:**

Sexual victimization (SV) is common among college women, with approximately half of those who have experienced SV meeting criteria for posttraumatic stress disorder (PTSD) within a year. Both SV and PTSD are associated with alcohol misuse among college women, often explained by the self-medication hypothesis. Existing literature focuses on overall PTSD severity rather than potential day-to-day fluctuations in specific symptoms, which might play a crucial role in understanding alcohol misuse risk. Studies also examine only same-day or next-day associations between PTSD and drinking, neglecting the potential for longer-term changes.

**Methods:**

This study explores the short-term longitudinal stability and time-lagged predictive dynamics of PTSD symptoms, affect, and drinking behavior among 174 female college heavy episodic drinkers over four weeks. Participants were categorized into three groups: those with a history of SV and PTSD *(n* = 77), women with SV but without PTSD (*n* = 59), and women without prior trauma history (*n* = 38) to be able to examine differences by trauma exposure, and PTSD. We compared the longitudinal stability of PTSD symptom networks, affect (arousal, positive affect, and negative affect), and drinking behavior across groups. Support vector regression determined which PTSD symptom networks and affect best predict drinking behavior at specific time lags within a 0-7 day range.

**Results:**

The PTSD group showed higher longitudinal stability for PTSD symptom networks (adjusted *p*s <.049) and arousal (adjusted *p*s <.048), but lower stability for negative affect (adjusted *p* =.013) and drinking behavior, including alcohol cravings (adjusted *p* =.019) and consumption (adjusted *p*s =.012), compared to the comparison groups. This suggests individuals with PTSD have more stable symptoms and arousal levels but greater fluctuations in negative affect and alcohol-related behaviors. Secondary analysis revealed PTSD symptom networks optimally predicted alcohol cravings with a three-day time lag (r=.88, *p* <.001) and consumption with a four-day time lag (r=.82, *p* <.001).

**Discussion:**

These findings challenge assumptions regarding immediate effects of PTSD and affect on drinking behavior and underscore the need for therapeutic approaches that consider longer-range effects. Future research should expand on these findings by incorporating longer-range assessments and exploring a broader range of symptom interactions.

## 1 Introduction

An estimated one in five women are sexually assaulted while attending college, exposing them to heightened risks of adverse mental health outcomes (Conley et al., 2017; Muehlenhard et al., 2017). Prospective studies have found that 41.9% of sexual assault survivors meet PTSD criteria one  year after the assault (Dworkin et al., 2023). A recent meta-analysis further concludes that individuals with a history of sexual assault have over seven times greater odds of developing PTSD compared to those with no such history (OR = 7.57; Dworkin, 2020). Child sexual abuse also increases risk of PTSD and increases risk of future victimization among college women (Messman-Moore et al., 2000). Sexual victimization and PTSD have been associated with increased risk of alcohol misuse (i.e., alcohol cravings and consumption) among college women, where each predicts the other over time (Read et al., 2012).

Survivors of sexual victimization (SV)—defined in this paper to encompass both childhood sexual abuse and sexual assault during adolescence and adulthood—also face challenges in regulating both positive and negative emotions (Berfield et al., 2022). Emotion dysregulation in PTSD can be seen via symptoms of persistent negative affect and difficulty experiencing positive affect (Hofmann et al., 2012; Berfield et al., 2022). Additionally, the college years are often a period of elevated and dynamic stressors that may cause fluctuations in mood (Zhang and Zheng, 2017; Barker et al., 2018).

College years and young adulthood are marked by high rates of alcohol consumption (Merrill and Carey, 2016; Krieger et al., 2018). Generally, college students tend to drink more often and more heavily than non-college-attending peers (Merrill and Carey, 2016). College drinking can be motivated by multiple factors including drinking to cope with negative affect, drinking to fit in, drinking to improve a good time or celebrate, and drinking as part of a social connection (Baer, 2002; Park and Levenson, 2002; Osberg and Boyer, 2018; King and Valley, 2019). As such, although individuals with sexual victimization histories, and those with PTSD, may drink to reduce symptoms or manage negative affect, they may also drink for other reasons as well (Huh et al., 2015; O’Donnell et al., 2019). College students also drink to cope with factors aside from PTSD including academic stress, negative mood, and affect variability (Gottfredson and Hussong, 2013; Peterson et al., 2021).

The prevailing explanatory theory behind relationships between SV, PTSD, and alcohol misuse is the self-medication theory, where alcohol is consumed as a means to cope with aversive symptoms of PTSD and to manage affect-related disturbances (Khantzian, 2003; Rhew et al., 2017; Dardis et al., 2021; Gilmore et al., 2024). There is ample cross-sectional, longitudinal, and micro-longitudinal or daily-level research supporting relationships between elevated PTSD symptoms and increased alcohol use and problems (Smith and Cottler, 2018; Hawn et al., 2020). Research that has focused on same-day or next-day relationships between PTSD symptoms and alcohol use has generally found that increased PTSD symptom severity or increased variance in PTSD symptom severity predicts same-day drinking (Cohn et al., 2014; Kaysen et al., 2014; Hruska et al., 2017; Wilson et al., 2017).

There are limitations to this general body of research. Many of the studies conducted to date use a PTSD total symptom severity score, which does demonstrate general positive associations between overall PTSD symptom severity and alcohol use (Hruska and Delahanty, 2012; Delker and Freyd, 2014; Tuliao et al., 2016; Eddinger et al., 2019). However, this may mask contributions from specific symptoms in relation to drinking. For example, in studies that have examined symptom clusters or individual symptoms some have found that re-experiencing symptoms or avoidance/numbing symptoms (Jakupcak et al., 2010; Lee et al., 2015) predict alcohol use but have not found those same relationships with arousal symptoms (Maguen et al., 2009; Jakupcak et al., 2010; Simpson et al., 2012; Hellmuth et al., 2013; Kaysen et al., 2014; Lee et al., 2015; Langdon et al., 2016). Among daily studies there have been several that have looked in more nuanced ways at how clusters or individual symptoms of PTSD relate to same-day or next-day drinking. Simpson et al. (2012) found that intrusive and avoidance symptoms were associated with next-day alcohol cravings in a small community sample. Kaysen et al. (2014) showed that female college students with elevated intrusive and behavioral avoidance symptoms experienced stronger urges to drink and were more likely to consume alcohol on those days. Sullivan et al. (2020) found that each PTSD symptom cluster was uniquely associated with concurrent alcohol consumption in women who had experienced intimate partner violence.

Affect also plays a critical role in understanding drinking behavior. Affect can be thought of as occurring along two dimensions, that of valence or pleasantness or unpleasantness and arousal or the level of activation and energy (Russell and Barrett, 1999; Posner et al., 2005). Two meta-analyses of non-clinical samples have found positive affect to be associated with near-term alcohol use, but no such association was found for negative affect (Tovmasyan et al., 2022; Dora et al., 2023). Among community members, higher positive affect was associated with increased alcohol consumption but only increased arousal and decreased variability in arousal predicted the likelihood of drinking (Peacock et al., 2015). Mean level of arousal predicted the quantity of alcohol consumed. In another community study, days with higher-than-usual levels of high arousal positive affect were associated with an increased odds of alcohol consumption and of heavy episodic drinking whereas there were no significant associations between high or low arousal negative affect and alcohol use (Jones et al., 2021). Conversely, research on women exposed to violence has shown that negative affect predicts same-day urges to drink and that affect regulation plays a crucial role in this link (Kaysen et al., 2007, 2014; Dyar and Kaysen, 2023; Stappenbeck et al., 2023).

These findings highlight the temporal and complex relationships between PTSD symptoms, affect, and drinking behavior, lending empirical support to the self-medication hypothesis. However, there remains a significant gap in the literature regarding the short-term longitudinal stability of PTSD symptoms, affect, and drinking behavior, such as day-to-day fluctuations over the course of a week or month. Research shows that PTSD symptoms are not constantly present among those with the diagnosis or subthreshold symptoms but instead can vary across time (McFarlane, 2000; Solomon et al., 2009) and even fluctuate widely from 1 day to the next (Black et al., 2016; Biggs et al., 2019; Schuler et al., 2021). The interactions among PTSD symptoms can be conceptualized as a short-term dynamic system to determine how variables affect one another over brief time intervals (Bringmann et al., 2023). To our knowledge, only one study to date has used a short-term dynamic model to examine PTSD symptoms (Greene et al., 2018), and no studies have yet assessed longitudinal changes in affect and alcohol misuse, nor the short-term dynamics between these measures. Thus, existing literature focuses on whether overall PTSD symptom severity or negative affect predict alcohol misuse, but does not test the role of fluctuations in PTSD severity or affect in understanding drinking. Understanding the role of these fluctuations could inform more precise interventions and help identify periods of increased vulnerability to alcohol misuse. This is the difference between whether people with higher PTSD or higher negative affect are at higher risk of alcohol misuse versus whether individuals who have more variability in their affect and symptoms have higher risk of alcohol misuse.

An additional limitation of the extant literature on relationships between PTSD and affect is the question regarding the time span within which these factors may influence drinking behavior. Existing micro-longitudinal studies examining links between PTSD-related symptoms and drinking behaviors have focused on PTSD or affect predicting drinking within the same assessment (so essentially micro-cross-sectional) or predicting the next assessment. Although there are theoretical justifications for examining same-day and next-day associations, it is equally important to investigate the extent to which changes in PTSD symptoms or affect may result in longer-term changes in drinking behaviors, as these may be mechanisms important in understanding development of alcohol use disorders over time. Without research addressing lagged effects, including longer term lagged effects, we cannot accurately validate our theoretical models concerning the temporal dynamics of how PTSD symptoms influence drinking behaviors.

### 1.1 Current investigation

Our study aims to build upon and complement prior research by exploring short-term longitudinal stability and time-lagged predictive dynamics of PTSD symptom networks, affect, and drinking behaviors (i.e., cravings and consumption). This study also examines the extent to which these relationships are a function of SV exposure versus PTSD by including college women with SV histories versus those without histories of trauma exposure, and by including women with and without probable PTSD. Our objective is to provide novel insights into the self-medication hypothesis for this specific population by describing how stability of symptom networks varies as a result of SV exposure *per se* and by further elucidating the triggers and timing of associations between symptom networks and alcohol use behaviors.

Instead of relying solely on aggregate measures of PTSD (i.e., total symptom or cluster scores), our investigation employs the state-of-the-art symptom network approach (Birkeland et al., 2020). Symptom network theory analyzes complex interactions among individual PTSD symptoms, illustrating how specific symptoms may influence or exacerbate one another. This method holds potential for uncovering the nuanced dynamics of PTSD, thereby deepening our understanding of its etiology, maintenance, and treatment possibilities. Instead of looking at summed scores which operationalize PTSD as a cohesive syndrome of symptoms, this approach acknowledges the heterogeneity in PTSD (DiMauro et al., 2014; Zoellner et al., 2014; Campbell-Sills et al., 2022; Bryant et al., 2023) and recognizes that various symptoms may differentially predict alcohol misuse. Conceptually, the symptom network approach views PTSD as having a graphical structure where symptoms are represented as ‘nodes’ (dots on a graph) and their relationships as ‘edges’ (lines connecting the dots), with each edge described by a ‘weight’ that signifies the strength of the correlation between symptoms. This framework allows for a detailed examination of the interdependencies and the overall structure of symptom relationships. For a comprehensive overview of the symptom network approach to PTSD, we refer the reader to Birkeland et al. (2020).

This is a secondary data analysis from an existing study focused on studying relationships between SV, PTSD symptoms, and affect in understanding alcohol misuse among college women (Kaysen et al., 2014). This study recruited college women who reported at least two occasions of binge drinking (i.e., four or more drinks on a single occasion) in the past month and had a history of SV, including childhood sexual abuse or sexual assault during adolescence or adulthood. The study included college women with recent heavy episodic alcohol use and at least subthreshold PTSD to ensure sufficient variability in key indicators when analyzing links between symptoms and alcohol use behaviors. This also allowed us to compare participants who met clinical criteria for probable PTSD to those who did not as means to isolate the effects of SV alone from those of PTSD symptoms. Lastly, the study included college women who engage in similar patterns of heavy episodic drinking but who had not been exposed to any potentially traumatic events to provide a comparison group that allows for differentiating between the effects of SV exposure and PTSD symptoms versus general college student behaviors. This comprehensive approach ensures that any observed differences in drinking behaviors can be more accurately attributed to the specific impacts of PTSD and trauma. By assessing these groups, we can determine if college students drink to regulate affect (both positive and negative) regardless of victimization or PTSD status, if they drink due to victimization histories independent of PTSD symptoms, or if they drink specifically because of PTSD symptomatology. The final sample (*n* = 174) thus included three distinct comparison groups: women with a history of SV and PTSD (“PTSD group”; *n* = 77), women with a history of SV but without PTSD (“SV-exposed group”; *n* = 59), and women without exposure to potentially traumatic events (“No-trauma group”; *n* = 38).

We conducted a secondary analysis on daily assessments from these 174 college women, in which they rated their PTSD symptomatology, affect, and alcohol behavior, including cravings and use, daily over the course of four weeks to address two primary goals:

Goal 1: To examine and compare the short-term, longitudinal stability of PTSD symptom networks, affect (i.e., arousal, positive affect, and negative affect), and drinking behavior (i.e., alcohol cravings and consumption) between the PTSD group and comparison groups.

Goal 2: To identify which PTSD symptom network (i.e., combinations of symptoms) and affective state features optimally predict alcohol cravings and consumption, over immediate and time-lagged intervals (i.e., ranging from 0 to 7 days to encompass a full week).

By addressing our two study goals, we aim to elucidate the mechanisms by which PTSD symptoms and affective states influence drinking behaviors over short-term periods, ultimately contributing to a deeper understanding of the self-medication hypothesis and informing clinical practices for this vulnerable population.

## 2 Materials and methods

The Institutional Review Board of the University of Washington approved all procedures, and we collected written consent from all participants.

### 2.1 Participants and procedures

#### 2.1.1 Screening and enrollment

**Figure 1:**
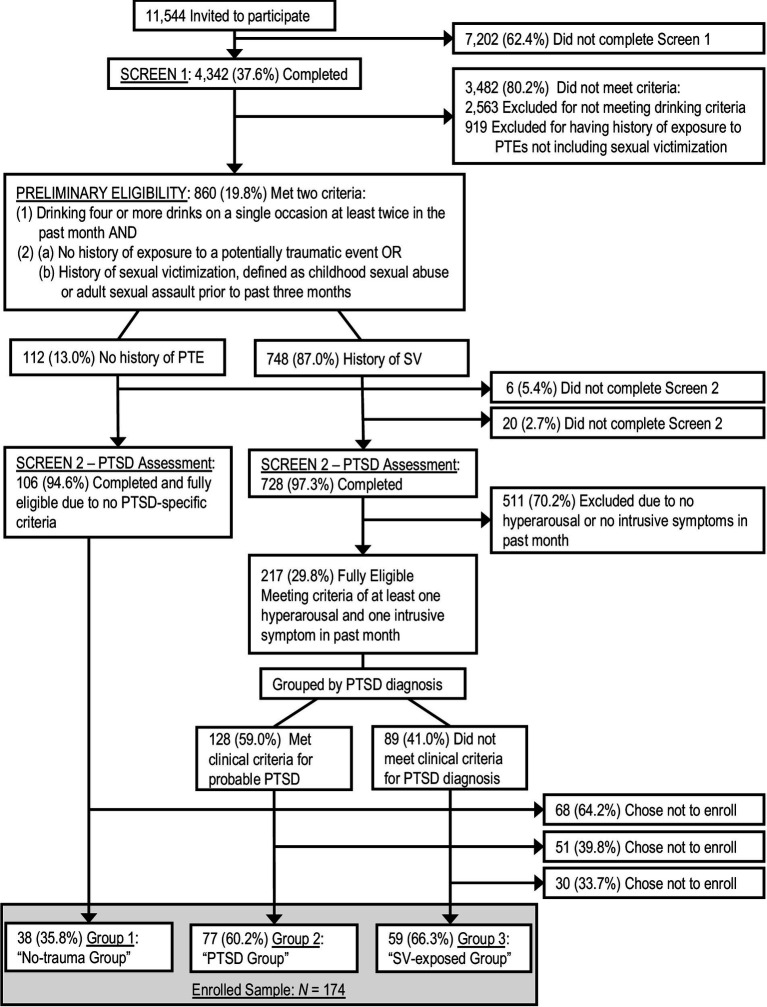
Flowchart of eligibility screening. We used following abbreviations: PTE, potentially traumatic event; SV, sexual victimization.

The full parent study details are described in (Kaysen et al., 2014). Briefly, we invited 11,544 randomly selected undergraduate women, via email and regular mail, to engage in a two-stage screening process designed to yield a sample comprised of the three distinct groups described above – women without exposure to any potentially traumatic event (“No-trauma group”), women with a history of SV and PTSD (“SV-exposed group”), and women with a history of SV but not meeting full PTSD criteria (“PTSD group“). This process, detailed in Figure 1, began with a 20-min online screening to assess preliminary eligibility: (1) two or more occasions of binge drinking – defined as four or more drinks on a single occasion (NIAAA, 2023) – in the prior month and (2) reporting *either* (a) no history of exposure to potentially traumatic events or (b) a history SV in the form of childhood sexual abuse or adult sexual assault that occurred prior to the past three months. The second screening assessed PTSD symptoms and provided a probable diagnosis via self-report measures described below. Full eligibility required that women who had a history of SV meet a third criteria: (3) endorsement of at least one intrusion and one hyperarousal PTSD symptom in the past month as an indicator of at least subthreshold PTSD. Results from the second screening were also used to divide SV-exposed participants into two groups based on probable PTSD. All individuals meeting full eligibility criteria (*n* = 323) were invited to schedule an in-person appointment at our study offices for training on study protocols and use of a study-provided personal digital assistant that would be used for data collection. Out of those invited, 174 women (55%) chose to schedule an appointment and enroll in the study – 38 in the “no-trauma group,” 77 in the “PTSD group,” and 59 in the “SV-exposed group.”

#### 2.1.2 Sample description

Participant ages ranged from 18 to 25. The mean age of study participants was 20.04 (SD = 1.35) years, with no between-group differences in age (Kruskal–Wallis test, *p* = 0.497; Table 1). A majority of participants identified as White (67.8%), while notable proportions represented Asian (13.2%), Multiracial backgrounds (8.0%), Latina (7.5%), African American (1.7%), Other (1.2%), and Native American (0.6%). Among participants with a history of SV, most had experienced sexual assault as an adult (93.4%), and nearly half had experienced child sexual abuse (44.9%).

**Table 1:** Description of the participant cohorts at baseline.

	**PTSD group**	**SV-exposed group**	**No-trauma group**	**Kruskal–Wallis test**
Number of participants	77	59	38	
Race				
*Asian*	9	7	7	
*African American*	3	–	–	
*Hispanic*	4	5	4	
*Native American*	1	–	–	
*White*	52	41	25	
*Multi-Ethnic*	8	4	2	
*Other*	-	2	–	
Age (*M ± SD*)	19.97 ± 1.32 years	20.22 ± 1.39 years	19.89 ± 1.73 years	*p* = 0.497
Peak drinking occurrences (*M ± SD*)	4.83 ± 2.92	4.56 ± 2.61	4.00 ± 2.05	*p* = 0.522
PTSD symptom severity (*M ± SD*)	40.42 ± 15.66	28.90 ± 8.89	24.95 ± 8.86	*p* < 0.001

The racial and ethnic composition across the three groups is comparable. Results show between-group differences in PTSD severity while we observed no differences in age nor peak drinking occurrences at baseline. We adjusted posthoc *p*-values using false discovery rate (FDR) correction for multiple comparisons.

#### 2.1.3 Data collection

The present study is a secondary analysis of data collected for a daily monitoring study of heavier drinking college women (Kaysen et al., 2014). Data collection for the parent study included two daily assessments – one before noon and another after 7:00 p.m. – over 30 consecutive days. However, a software error resulted in the absence of data for the final day, resulting in continuous daily data spanning 29 days. While meeting with study staff upon enrollment, a research assistant trained participants on how to use the handheld personal digital assistant data collection devices and how to respond to the survey prompts. Alerts from the device sounded at times of the day selected by each participant to indicate the start of a two-hour assessment window. Assessments required four min to complete, on average. Compensation included $45 for screening and baseline assessments, $1 for each completed daily assessments, as well as bonuses for completion of consecutive assessments of various intervals. Potential earnings for completion of all assessments totaled $215.

Assessments scheduled for completion prior to noon included all variables of interest in the present study – PTSD symptoms, affect, alcohol use, and cravings – and are included in analyses. Participants completed 68.9% of morning assessments, yielding 3,597 instances of data across 174 individuals available for analyses. Over the 30-day window, the sample median days of data completion was 23. The completion rate is similar to other micro-longitudinal studies of PTSD and alcohol use (e.g., Cohn et al., 2014; Hruska et al., 2017; Black et al., 2018).

### 2.2 Screening and baseline assessments

#### 2.2.1 Alcohol consumption

The Quantity Frequency Questionnaire (Dimeff, 1999) served as an assessment tool for peak drinking occurrences within the last month. Inclusion criteria for participants encompassed those who acknowledged consuming four or more drinks on at least two occasions in the past month. A drink was specified as 12 oz. of beer, 10 oz. of microbrew or wine cooler, 4 oz. of wine, or one cocktail with 1 oz. of 100-proof liquor or 1.25 oz. of 80-proof liquor. There were no significant differences in peak drinking occurrences between the groups (Kruskal–Wallis test, *p* = 0.522; Table 1).

#### 2.2.2 Traumatic exposures

Exposure to potentially traumatic events were assessed using the Traumatic Life Experiences Questionnaire (Kubany et al., 2000). Participants were asked to report whether they had ever experienced each of 17 Criterion A events. For each endorsed event, follow-up questions established whether fear, horror, helplessness, or physical injury were experienced as a result of the event, as is necessary for meeting the DSM-IV definition of a Criterion A event (American Psychiatric Association, 1998). Prospective participants exposed to a potentially traumatic event other than sexual victimization were deemed ineligible.

#### 2.2.3 Sexual victimization

We assessed SV in the form of childhood sexual abuse or adult sexual assault occurring prior to the past three months via the Childhood Victimization Questionnaire (Finkelhor, 1979) and Sexual Experiences Survey (Koss and Oros, 1982; Koss and Gidycz, 1985). Childhood sexual abuse was defined as “any sexual activity perceived as coercive or forced that occurred before the age of 14 with someone five or more years older.” Participants were asked to report any of the 11 unwanted sexual experiences, ranging from a sexual invitation to intercourse, using response options 1 for “yes” and 0 for “no.” Adolescent and adult sexual assault was characterized as any “unwanted oral-genital contact, vaginal/anal intercourse, and/or penetration by objects since the age of 14.” This encompassed both attempted and completed instances of unwanted oral, vaginal, and anal sexual intercourse. Participants responded to 18 experiences, indicating 1 for “yes” and 0 for “no.”

#### 2.2.4 PTSD diagnosis and symptom severity

To assess PTSD symptomatology for study inclusion, we used the Posttraumatic Diagnostic Scale developed by Foa et al. (1997). Participants indicated how much each PTSD symptom had bothered them in the past month. For women with histories of SV, the focus was on their most distressing unwanted sexual experience, while women with no trauma histories were instructed to concentrate on their most stressful life event. Response options ranged from 0 (not at all) to 3 (very much) on a Likert scale. For the purpose of defining comparison groups, probable PTSD diagnostic status was determined by meeting Criteria B (1 intrusive symptom), C (3 avoidance symptoms), and D (2 hyperarousal symptoms) of the DSM–IV (American Psychiatric Association, 1998) based on self-report. As expected, the PTSD group showed highest total PTSD symptom severity [Kruskal–Wallis test, χ^2^(2) = 46.331, *p* < 0.001; Table 1], surpassing both SV-exposed (adjusted *p* < 0.001) and no-trauma groups (adjusted *p* < 0.001). The SV-exposed group also exhibited higher PTSD symptom severity than the no-trauma group (adjusted *p* = 0.023). We corrected for multi comparisons using false discovery rate (FDR; Benjamini and Hochberg, 1995).

### 2.3 Daily monitoring assessments

#### 2.3.1 PTSD symptom severity

The PTSD Checklist Specific version (PCL-S; Wilkins et al., 2011) includes 17 items assessing PTSD symptoms defined in Criteria B, C, and D of the DSM–IV (American Psychiatric Association, 1998). Women with SV histories focused on their most distressing unwanted sexual encounters, while those without trauma focused on a significant stressful life event. Modified for daily assessments (Naragon-Gainey et al., 2012), participants indicated how much each symptom bothered them within the past 24 h. Responses ranged on a Likert scale of 1 (not at all) to 5 (extremely). Internal consistency of the measure was very strong in the current sample (Nezlek’s alpha = 0.92; Nezlek, 2017).

#### 2.3.2 Affect

We utilized a modified version of the Positive and Negative Affect Scale (PANAS; Watson et al., 1988) to evaluate participants’ affect states. Participants were presented with various emotions and asked to indicate their feelings “at the present moment.” Affect items, selected from the circumplex model of affect, encompassed *arousal* (i.e., tired [reversed], calm [reversed], tense), as well as positive (i.e., happy, delighted) and negative valanced words (i.e., angry, bored, miserable, sad; Russell, 1980; Remington et al., 2000). Response options ranged from 1 (“no!!”) to 4 (“yes!!”). Nezlek’s alphas suggested robust internal consistency, registering at 0.71 for arousal, 0.73 for positive affect, and 0.72 for negative affect.

#### 2.3.3 Alcohol cravings

Participants responded to the subsequent statements to assess their inclination to drink since the previous evaluation: (a) “I really have not felt like drinking,” (b)” I felt like I could really use a drink,” and (c) “The idea of drinking has been appealing.” Using a Likert scale ranging from 0 (“definitely false”) to 8 (“definitely true”), participants indicated their agreement with each statement. We summed the items to create the daily alcohol craving measure, with the initial statement being reverse scored. The items exhibited strong internal consistency in the present sample, with Nezlek’s alpha measuring 0.89.

#### 2.3.4 Alcohol consumption

Queries about alcohol consumption focused on participants’ usage in the preceding 24 h. We prompted participants with the question, “How many standard drinks have you had in the past 24 h?” A standard drink was defined as 12 oz. of beer, 10 oz. of microbrew or wine cooler, 4 oz. of wine, or 1 cocktail with 1 oz. of 100-proof liquor or 1.25 oz. of 80-proof liquor. Participants could input the number of drinks directly into the personalized digital assistant. For those who abstained from alcohol, the option was available to either type in “0″ or select the response “I did not drink.”

### 2.4 Statistical analysis

#### 2.4.1 Assessment of longitudinal stability of PTSD symptom networks

Our first goal was to examine and compare how stable patterns of PTSD symptoms were over time in the PTSD group versus the comparison groups. To accomplish our objective, we applied a commonly applied approach in symptom network analyses called Gaussian graphical modeling (Armour et al., 2017; Epskamp et al., 2018; Birkeland et al., 2020). These models pinpoint each symptom as a node of a graph and elucidate how symptoms are interconnected by treating the associations between them as links, thereby revealing the underlying structure of the symptom network. Centrality measures such as strength, betweenness, and closeness can be used for comparative analyses (Armour et al., 2017; Epskamp et al., 2018; Birkeland et al., 2020). In this study we focused on the 17 symptoms from the DSM-IV criteria (American Psychiatric Association, 1998), which include (1) intrusive memories, (2) nightmares, (3) flashbacks, (4) distress at reminders, (5) physiological arousal at reminders, (6) avoidance of thoughts/feelings, (7) avoidance of activities and situations, (8) psychogenic amnesia, (9) loss of interest, (10) emotional isolation, (11) emotional numbing, (12) foreshortened future, (13) sleep disturbance, (14) irritability, (15) concentration problems, (16) hypervigilance, and (17) excessive startle. We adopted the Gaussian graphical model procedures outlined by Von Stockert et al. (2018) to derive daily PTSD symptom networks. In other words, we calculated Spearman’s correlations for all possible pairs of symptoms (136 pairs) on a daily basis – across all participants of the same group. This is commonly followed by the extended Bayesian information criterion (EBIC) Glasso methodology (Friedman et al., 2008; Foygel and Drton, 2010) to eliminate connections with small associations. However, we recognize that the EBIC Glasso methodology tends to result in an unconnected graph for smaller sample sizes. We therefore used *p* > 0.05 as more stable criterion to eliminate insignificant connections. To assess longitudinal stability across daily symptom networks (characterized by 136 daily correlations between symptom pairs), we employed a heteroscedastic linear Gaussian model (Carroll and Ruppert, 1982). This model assumes that each correlation for each symptom pair at each day follows a normal distribution, with the mean and standard deviation adhering to an affine function: (1) the *temporal expectation of the model mean [μ]* (i.e., the average predicted correlation value at day 15), (2) the *temporal change of the model mean [Δμ]* (i.e., the predicted correlation slope indicating mean changes over time), (3) the *temporal expectation of the model’s standard deviation [σ]* (i.e., the average predicted standard deviation at day 15), and (4) the *temporal change of the model’s standard deviation [Δσ]* (i.e., the predicted standard deviation slope indicating changes over time). This simplified surrogate model offers the advantage of straightforward interpretability over utilizing a (potentially incomplete) time series of correlation values. The estimated models, along with visualizations of the four performance metrics, are presented in Supplementary Figures S1–S3. While the first performance metric *[μ]* provides descriptive insights into correlation scores between PTSD symptoms, metrics *Δμ*, σ, and *Δσ* capture different aspects of longitudinal variance, offering essential information for evaluating longitudinal stability.

To ensure the validity of our analyses, we first calculated the mean total PTSD symptom severity (i.e., the summed score across all 17 PTSD symptoms) for each participant over the four weeks. We then compared these mean scores between the groups using a Kruskal–Wallis test to confirm that the PTSD group exhibited greater symptom severity. Post-hoc multiple comparisons were FDR-corrected, and the corresponding boxplots are provided in Supplementary Figure S4. We then compared the longitudinal stability of PTSD symptom networks across participant groups – individuals with PTSD versus two comparison groups. Specifically, we conducted Kruskal–Wallis tests to explore potential differences among the three groups (PTSD group, SV-exposed group, no-trauma group) for each of the four metrics (*μ, Δμ, σ*, and *Δσ*). To mitigate the impact of multiple comparisons, we applied FDR correction to the obtained *p*-values. This correction accounted for multiple testing across the four metrics and subsequent posthoc analyses within each metric (i.e., three tests).

#### 2.4.2 Assessment of longitudinal stability of affect and drinking behavior

We also sought to assess and contrast the short-term, longitudinal stability of affect (i.e., arousal, positive affect, and negative affect) and drinking behavior (i.e., alcohol cravings, alcohol consumption) between the PTSD group and the two comparison groups. Given the distinct data structure (i.e., raw questionnaire data) of the affect and drinking behavior measures compared to the symptom network data (where we had 136 correlation values per group and metric), we needed to introduce complementary longitudinal stability metrics. This adjustment was necessary to align with the structure outlined in the previous section (2.4), particularly when employing heteroscedastic linear Gaussian models to predict trends. The objective was to ensure consistency in the evaluation of longitudinal stability across different measures. Specifically, we calculated (1) the *mean scores [*ν*]* of arousal, positive affect, negative affect, alcohol cravings, and alcohol consumption over the 29 days to provide a baseline assessment of the central tendency of the data. We then introduced three metrics to assess the longitudinal stability of the five measures: (2) the *absolute (day-to-day) mean difference [Δ*ν*]* to assess how much the central tendency of the data changes from 1 day to the next (i.e., a smaller absolute difference indicates more stability in the central tendency over time, suggesting that the measure tends to stay relatively constant); (3) the *standard deviations [τ]* over the 29 days to provide insights into the variability or spread of your data across days (i.e., a consistent standard deviation across days suggests stability in the dispersion of your data, while significant changes may indicate fluctuations in variability); and (4) the *absolute (day-to-day) standard deviation differences [Δτ]* to assess how much the standard deviation varies from 1 day to the next without considering the direction of change (i.e., a smaller absolute difference implies greater stability in the variability of your measure over the observed period). We provide a visualization of the four metrics in Supplementary Figures S5, S6.

To avoid violating the independence assumption inherent in Kruskal–Wallis tests, we first conducted permutation tests on the median values between the PTSD group and the combined comparison groups (SV-exposed and no-trauma controls) for each of the five measures (i.e., arousal, positive affect, negative affect, alcohol cravings, and alcohol consumption), running 100,000 permutations for each test. We applied FDR corrections for multiple testing across the four metrics within each measure. If a significant difference was found, we proceeded with post-hoc permutation tests to compare medians across all groups (PTSD, SV-exposed, and no-trauma controls), again correcting for multiple comparisons (i.e., three tests).

#### 2.4.3 Predicting longitudinal dynamics of drinking behavior

The second goal of this study aimed to identify key variables within the longitudinal trajectories of PTSD symptom networks (136 features at each time step) and affect (3 features at each time step) for predicting drinking behavior trajectories within the PTSD group. The specific objective was to pinpoint the most predictive feature combinations, considering a time lag ranging from 0 to 7 days (to account for both immediate and time-lagged associations), to predict both alcohol cravings and consumption. We used support vector regression (Drucker et al., 1996) with a radial basis function kernel for prediction (Hastie et al., 2009), focusing on identifying the optimal features for predicting alcohol cravings and consumption. Due to computational limitations, we considered feature combinations of 1, 2, and 3 features. The exhaustive feature search covered combinations of 1, 2, and 3 features, with a total of 139, 9.591, and 437.989 combinations, respectively. In other words, the algorithm first looked for the single feature that predicts drinking behavior best with a time lag of 0 days, then 1 day, and so on up to 7 days. It then did the same for combinations of two features, and finally for combinations of three features. This assessment allowed us to determine whether 1, 2, or 3 features and what time lag would provide the highest prediction for alcohol cravings and consumption. To address overfitting, we conducted a leave-one-out cross-validation procedure (Hastie et al., 2009), iteratively evaluating model performance and selecting the best features based on the ratio of the absolute Spearman correlation to the mean squared error. This process resulted in a set of one, two, or three features for each time lag from 0 to 7 days for both alcohol cravings and consumption targets. In the final feature selection, we considered the absolute ratio of the Spearman correlation to the mean square error across features and time lags as the optimization criterion. Put simply, the final choice was made from all combinations based on whichever had the highest ratio score, indicating the highest correlation and the lowest error rate.

## 3 Results

We conducted all statistical analyses in SPSS Statics 28. We initially conducted a Kruskal–Wallis test to assess potential variations in completions rates for daily assessments among the three groups. Results did not reveal significant differences in completion rates among the three groups (*p* = 0.295). Completion rates were comparable, with the PTSD group at 68.2 ± 0.25%, the SV-exposed group at 69.3 ± 0.27%, and the no-trauma group at 75.0 ± 0.24%. We proceeded with the main data analysis.

### 3.1 Longitudinal stability of symptom networks

**Figure 2:**
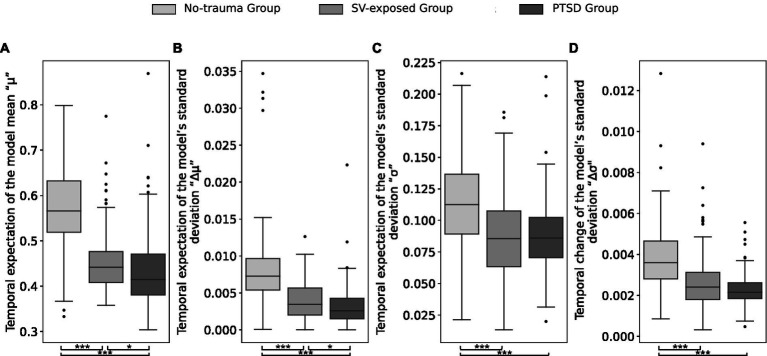
Results of the symptom network longitudinal stability analysis. Between-group differences for the four metrics including the temporal expectation of the model mean “μ” **(A)**, the temporal change of the model mean “Δμ” **(B)**, the temporal expectation of the model’s standard deviation “σ” **(C)**, and the temporal change of the model’s standard deviation “Δσ” **(D)**. Statistically significant differences are denoted with *** at FDR-corrected *p* < 0.001 and * at *p* < 0.05.

The validation analysis confirmed significant differences in total PTSD symptom severity between the groups [χ^2^(2) = 50.019, *p* < 0.001]. The PTSD group exhibited the highest PTSD symptom severity (adjusted *p* < 0.001), followed by the SV-exposed group, which had higher severity than the no-trauma group (adjusted *p* = 0.02). Based on these confirmed differences, we proceeded with longitudinal stability analyses. Results revealed significant group differences for *temporal expectation of the model mean “μ”* (χ^2^(2) = 164.66, adjusted *p* < 0.001), *temporal change of the model mean “Δμ”* [χ^2^(2) = 118.20, adjusted *p* < 0.001], *temporal expectation of the model’s standard deviation* “*σ*” [χ^2^(2) = 52.482, adjusted *p* < 0.001], and *temporal change of the model’s standard deviation “Δσ”* [χ^2^(2) = 102.51, adjusted *p* < 0.001; Figure 2]. Posthoc pairwise comparisons, incorporating FDR correction, indicated that the PTSD group had significantly lower values for variables *μ* (adjusted *ps* < 0.049), *Δμ* (adjusted *p*s < 0.018), and *Δσ* (adjusted *p*s < 0.038) compared to the SV-exposed and the no-trauma groups. Variable *σ* indicated lower scores for the PTSD group compared to the no-trauma group (adjusted *p* < 0.001), with no significant difference between the PTSD group and SV-exposed group (adjusted *p* = 0.938). The SV-exposed group consistently exhibited significantly lower values than the no-trauma group for all four metrics (*μ, Δμ, σ, Δσ;* adjusted *p*s < 0.001). It is important to note that the first metric (graph A in Figure 2), the temporal expectation of the model mean “μ,” is included for the purpose of analytic transparency and should not be over-interpreted. Due to the skewed nature of the PTSD symptom ratings – specifically, the lower variance in responses among the no-trauma and SV-exposed groups, who predominantly reported lower scores on a 1–5 scale (see histograms in Supplementary Figure S4B) – correlation values between these two groups are therefore inflated for this measure. This potential inflation does not impact the other three metrics, which offer a clearer insight into the temporal dynamics of PTSD symptoms. The results overall suggest reduced longitudinal variance (i.e., increased longitudinal stability) within the PTSD group, followed by the SV-exposed group, and the highest variance observed in the no-trauma group. These findings underscore the differential stability of PTSD symptomatology across groups, with the PTSD group demonstrating the most pronounced longitudinal stability.

### 3.2 Longitudinal stability of affect and drinking behavior

Analyses using permutation tests indicated significant differences in median scores between the PTSD group and the combined comparison groups for arousal (adjusted *p* < 0.001), positive affect (adjusted *p* = 0.014), negative affect (adjusted *p* < 0.001), and alcohol cravings (adjusted *p* = 0.009) while no significant differences were found for alcohol consumption (adjusted *p* = 0.707). Post-hoc pairwise comparisons revealed that the PTSD group exhibited the highest arousal (adjusted *ps* < 0.001) and negative affect scores (adjusted *ps* < 0.001) and the lowest positive affect scores (adjusted *ps* < 0.044) compared to both comparison groups. Additionally, the PTSD group had higher alcohol cravings compared to the SV-exposed group (adjusted *p* = 0.004) and the no-trauma group, although the latter did not reach significance after correction (adjusted *p* = 0.158). The data trends revealed that individuals with PTSD displayed higher scores for arousal, negative affect, and alcohol cravings, and lower scores for positive affect.

**Figure 3:**
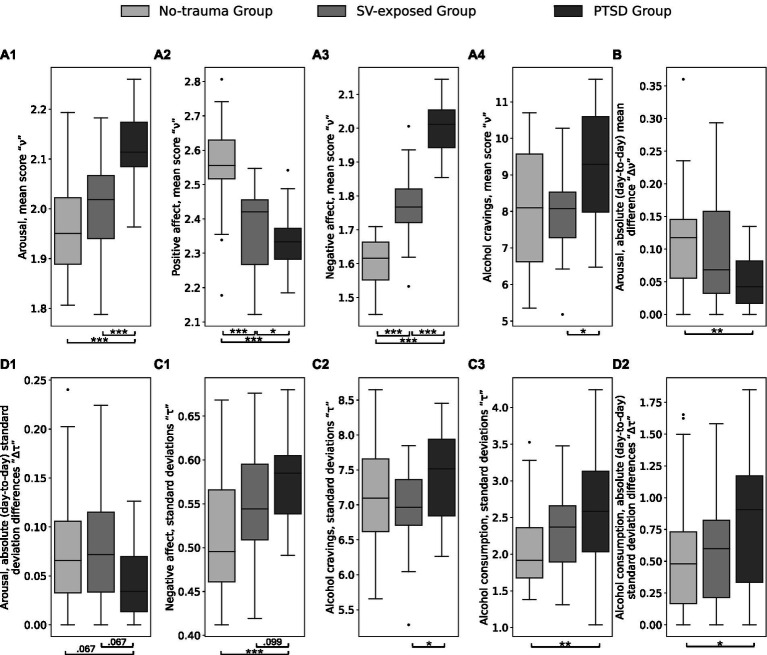
Results of the longitudinal stability analysis for the affect and drinking behavior. Between-group differences for the four metrics including (1) the mean scores *“*ν*”* **(A1–A4)**, (2) absolute (day-to-day) mean difference *“Δ*ν*”* **(B),** (3) standard deviations *“τ*” **(C1–C3)**, (4) and absolute (day-to-day) standard deviation differences “*Δτ*” **(D1,D2)**. Statistically significant differences are denoted with *** at FDR-corrected *p* < 0.001, ** at *p* < 0.01, and * at *p* < 0.05.

In terms of longitudinal stability, the PTSD group showed significant differences in two metrics for arousal: absolute mean differences *“Δ*ν*”* (adjusted *p* = 0.048) and absolute standard deviation differences “*Δτ*” (adjusted *p* = 0.048) compared to the combined comparison group. Specifically, the PTSD group had lower absolute mean differences in arousal compared to the no-trauma group (adjusted *p* = 0.002), but not the SV-exposed group (adjusted *p* = 0.376). Absolute standard deviation differences in arousal were also lower for the PTSD group compared to both comparison groups, though not significantly after multiple comparisons (adjusted *ps* = 0.067). Significant differences were also found between the PTSD group and the combined comparison groups in the standard deviation *“τ*” for negative affect (adjusted *p* = 0.013), with the PTSD group showing higher variance compared to the no-trauma group (adjusted *p* < 0.001), and a non-significant trend compared to the SV-exposed group (adjusted *p* = 0.099). For alcohol cravings, significant differences in standard deviation *“τ*” were observed between the PTSD group and the combined comparison groups (adjusted *p* = 0.019), with the PTSD group exhibiting higher values compared to the SV-exposed group (adjusted *p* = 0.041) and the no-trauma group, though the latter did not remain significant after correction (adjusted *p* = 0.175). Regarding alcohol consumption, significant differences were noted in standard deviation *“τ*” (adjusted *p* = 0.012) and absolute standard deviation differences *“Δτ”* (adjusted *p* = 0.012) between the PTSD group and the combined comparison groups. The PTSD group had higher values for both metrics compared to the no-trauma group (adjusted *ps* < 0.037) and the SV-exposed group, though the latter did not reach significance after correction (adjusted *ps* > 0.189). No significant differences were found for the longitudinal stability of positive affect (adjusted *ps* > 0.128). Figure 3 summarizes the results. The findings indicate notable variations in longitudinal stability, with the PTSD group exhibiting lower longitudinal variance (i.e., higher longitudinal stability) for arousal, and higher longitudinal variance (i.e., lower longitudinal stability) for negative affect and drinking behavior, including alcohol cravings and consumption.

### 3.3 Predicting longitudinal dynamics of drinking behavior

**Figure 4:**
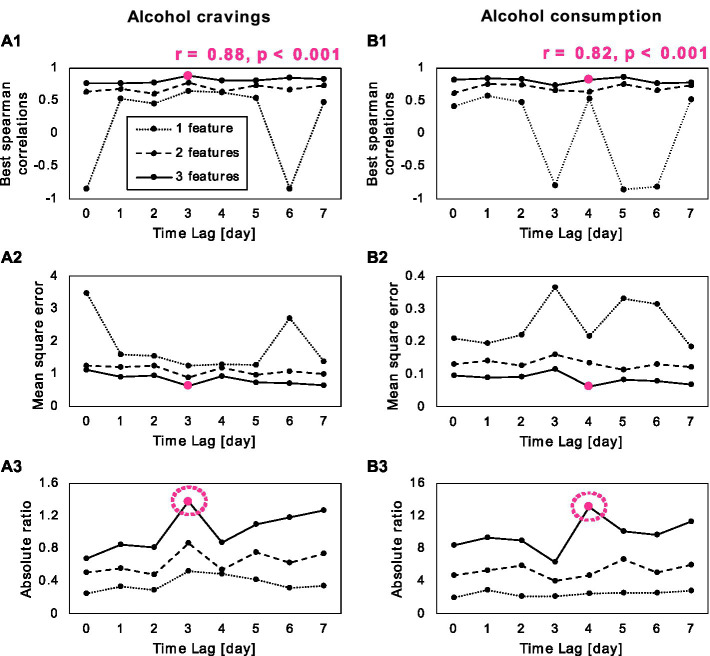
Results of the second prediction analysis for **(A)** alcohol cravings and alcohol consumption **(B)**. **(A1,B1)** Correlation between the target and predicted scores, across different feature numbers (1–3) and different time lags (0–7 days). **(A2,B2)** Shows the mean square errors of the model across different feature numbers and time lags. **(A3,B3)** Depicts the absolute ratio between correlation and mean square error across different feature numbers and time lags, with an optimal feature number of three with a 3-day time lag for alcohol cravings and 4-day time lag for alcohol consumption (pink circle). The optimal feature set included symptom correlations between intrusive memory and concentration problems, distress at reminders and physiological arousal at reminders, and emotional numbing and sleep disturbance for alcohol cravings. For alcohol consumption, the feature set included symptom correlations between intrusive memories and irritability, intrusive memories associated and hypervigilance, and psychogenic amnesia correlated and loss of interest.

The process of our predictive model training is illustrated in Figure 4, in which Spearman correlation coefficients between target and predicted scores, mean square error, and the absolute ratio between correlation coefficient and mean square error are presented across different feature sets and time lags. The optimal combination of feature set and time lag was indicated by the highest absolute ratio value (i.e., highest absolute correlation coefficient with lowest error; pink circles in Figures 4A3,B3). For alcohol cravings, the results revealed the most effective prediction of alcohol cravings occurred with three features and a three-day time lag, demonstrating a robust correlation (*r* = 0.88; *p* < 0.001). This optimal feature set included symptom correlations between intrusive memory and concentration problems, distress at reminders and physiological arousal at reminders, and emotional numbing and sleep disturbance. In the context of alcohol consumption, our results indicate that the best prediction involved three features with a four-day time lag (*r* = 0.82, *p* < 0.001). These features included symptom correlations between intrusive memories and irritability, intrusive memories associated and hypervigilance, and psychogenic amnesia correlated and loss of interest.

## 4 Discussion

The overall goal of this study was to explore the impact of SV on short-term, longitudinal stability and immediate and time-lagged predictive dynamics of PTSD symptoms, affect, and drinking behaviors among college women. To address this goal, we first compared the longitudinal stability of these measures across three groups: college women with a history of SV and PTSD, those with a history of SV but no PTSD, and those with no history of either. We then applied support vector regression to determine which feature set of PTSD symptom networks and affect best predicts drinking behavior at the optimal specific time lag within the 0–7 day range.

Prior to addressing goals 1 and 2, we conducted validation analyses to confirm anticipated data trends regarding PTSD symptoms, affect, and drinking behavior among the groups. As expected, our findings indicated significant group differences, with the PTSD group showing the highest mean levels of PTSD symptoms, arousal, negative affect, and alcohol cravings, and the lowest levels of positive affect compared to the SV-exposed and no-trauma groups. These findings confirm previous research that has highlighted the emotional and psychological impact of PTSD on individuals (Peter-Hagene and Ullman, 2015; Straus et al., 2018). As is consistent with extant literature, PTSD is associated with higher arousal, negative affect, and alcohol cravings, as compared to SV exposure alone, and compared to college women without prior trauma exposure. SV also has an effect, but less than SV coupled with PTSD.

The first goal of this study was, in part, to examine the longitudinal stability of PTSD symptom networks, comparing across those with PTSD, those with sexual victimization but without PTSD, and those without a trauma history. Looking at the stability of these networks across these groups allows us to test whether these symptoms are more chronic or more transient based on SV history and probable diagnosis. Results indicate greatest longitudinal stability of PTSD symptom networks among participants with probable PTSD, with lowest variance across the metrics for women in the PTSD group and highest variance for the no-trauma controls. Consistent with prior research showing stability of PTSD symptom networks over longer periods, ranging from six months to five years (Von Stockert et al., 2018; Ge et al., 2019; Crowe et al., 2023), our findings are the first to show micro-longitudinal stability of PTSD symptoms using a network analysis approach. In contrast, using linear mixed models, Biggs et al. (2019) compared daily fluctuations in PTSD symptom severity across two groups of U.S. military personnel – those with and without probable PTSD. Results showed that among personnel with probable PTSD, symptoms were more severe on weekdays relative to weekends. No such variance was observed among those without probable PTSD suggesting more variance for those with PTSD as compared to more stability as was found in our study. These contradictory findings may be attributable to differences in methodology (i.e., analytic approach and/or operationalization of the disorder), college women as compared to military personnel, type of trauma exposure, or the presence of binge drinking. For example, the authors note that the weekday context of working in a military environment may expose service personnel to frequent reminders of military-related traumas. Conversely, college women with sexual assault histories may encounter less day-dependent, reminders of their traumatic experiences. Given the limited amount of research in this area, and the differences in findings across these two studies, replication is warranted. Our results suggest that the temporal stability of symptoms among individuals with PTSD can be attributed to the persistent and enduring nature of the disorder, whereas individuals without trauma histories exhibit greater fluctuation in symptom correlations, indicating that these responses may be indicative of momentary distress to life stressors.

Additionally, the PTSD group exhibited greater longitudinal stability in arousal and greater longitudinal variance in negative affect compared to both comparison groups, indicating significant challenges in emotional regulation. This pattern aligns with existing literature that links PTSD to emotional dysregulation (Weiss et al., 2020; Haws et al., 2022). The combination of stable, persistent arousal symptoms and fluctuating negative affect highlights a complex emotional profile, consistent with previous research associating the arousal cluster with emotional dysregulation (Weiss et al., 2020; Haws et al., 2022). Notably, difficulty in maintaining persistent arousal and regulating mood (negative affect) is associated with maladaptive coping strategies such as alcohol use (Lee et al., 2015; Von Stockert et al., 2018; Ge et al., 2019; Haws et al., 2022; Crowe et al., 2023).

With respect to drinking behavior, our findings showed that the PTSD group had higher longitudinal variance in alcohol cravings and consumption compared to the other groups. These findings suggest that individuals with PTSD experience greater longitudinal variability in alcohol cravings and consumption. This variability suggest that individuals with PTSD may crave and use alcohol as a coping mechanism to manage their persistent PTSD symptoms and arousal, as well as fluctuating mood states (Bolton et al., 2009; Turner et al., 2018). Our combined findings of longitudinal stability of PTSD symptoms and arousal, higher longitudinal variance of negative affect (mood), and higher longitudinal variance of alcohol cravings and use indicate a complex interaction between stable distress and fluctuating mood states in individuals with PTSD. This pattern underscores the chronic and multifaceted nature of PTSD, where persistent symptoms are coupled with attempts to self-medicate through alcohol use to manage emotional dysregulation. These insights highlight the need for targeted interventions that address both the enduring symptoms of PTSD and the maladaptive coping strategies employed by individuals to alleviate their emotional distress.

Regarding our second goal, we found that alcohol cravings and consumption were best predicted by unique PTSD symptom network features (i.e., correlated sets of PTSD symptoms) experienced 3–4 days prior. Specifically, intrusive memories, concentration problems, distress at reminders, physiological arousal at reminders, emotional numbing, and sleep disturbance optimally predicted alcohol cravings 3 days later. Intrusive memories, irritability, intrusive memories, hypervigilance, psychogenic amnesia, and loss of interest best predicted alcohol consumption 4 days later. Notably, four of the six factors predicting alcohol consumption or cravings included at least one symptom from the re-experiencing cluster. This is consistent with existing research demonstrating associations between re-experiencing symptoms and drinking outcomes (Simpson et al., 2012; Kaysen et al., 2014; Sullivan et al., 2020). Unlike these previous studies, which found immediate (i.e., same or next assessment) effects, our results highlight the significance of time-lagged associations, particularly with arousal-cluster symptoms. The finding that certain PTSD symptom interactions may have a delayed effect on drinking behavior has important implications for treatment and relapse prevention strategies. For example, “urge surfing” is such a strategy used to manage cravings. The underlying model conceptualizes alcohol cravings and usage to cycle over short periods, typically within minutes to hours (Ostafin and Marlatt, 2008; Hisler et al., 2022). The strategy therefore encourages its users to ride out the wave of discomfort associated with an unmet urge to drink, suggesting that it will be short-lived. However, our findings suggest that some PTSD symptoms may have lingering effects on alcohol cravings and use. Similarly, in relapse prevention, clinicians work with clients to identify immediate triggers for potential use, but these findings suggest that the triggers may be more temporally distal. Ongoing research is needed to confirm and further explore dynamics of time-lagged effects within the self-medication hypothesis. For example, future research should test whether the stress of symptoms accumulates over a period of time, then resulting in drinking behaviors. Regardless of causal mechanisms, if confirmed, present findings may suggest that clinicians should work with clients regarding managing not only day-to-day stressors as antecedents of drinking but also addressing how to manage lingering effects of those stressors over time for those with PTSD.

Finally, while the PTSD group experienced higher alcohol cravings, there were no significant differences in actual alcohol consumption across the groups. This suggests that although individuals with PTSD have stronger urges to drink, these cravings do not necessarily result in increased consumption. Higher cravings may reflect an ongoing desire to use alcohol as a coping mechanism, consistent with the self-medication hypothesis, but actual drinking behavior may be influenced by situational constraints and individual differences in managing these urges.

Our choice of cohort, which included only individuals who already engage in heavy episodic drinking, may also contribute to the observed lack of differences in drinking consumption, as it did limit the sample to students already engaging in some high-risk drinking. However, this specific cohort provides unique insights into how trauma and PTSD symptoms specifically influence cravings within a context where high levels of drinking are more prevalent. Our results suggest that cravings may reflect underlying psychological distress and motivations for drinking that differ from actual consumption levels. Future research should further assess these dynamics to understand the underlying mechanisms and to develop targeted interventions.

Our findings on the longitudinal stability and time-lagged effects of PTSD symptom networks and effect on alcohol cravings and consumption have significant implications for interventions targeting college women exposed to SV. Understanding the specific PTSD symptoms and affective states that predict drinking behavior can lead to more targeted and effective therapeutic approaches. Cognitive-behavioral therapies that incorporate emotion regulation training and exposure therapy may help individuals develop healthier coping mechanisms and reduce their reliance on alcohol (Messman-Moore and Ward, 2014). Findings generally support the use of integrated trauma-focused substance use treatment, such as COPE as the skills can address reducing both the PTSD and managing alcohol cravings (Mills et al., 2012; Back et al., 2014). By focusing on the underlying emotional and psychological factors driving alcohol use, these interventions can provide more sustainable and effective outcomes.

Future studies should also develop computational methods to consider more than three features, which could reveal more complex interactions between symptoms and drinking behavior. While our current approach focuses on the three most important symptom correlations due to computational limitations, this strategy also ensures that therapy can be effective by prioritizing the most impactful factors. This dual focus on computational efficiency and clinical relevance highlights a promising direction for future research and therapeutic development.

The current investigation has several limitations that warrant consideration. First, analyzed data on PTSD and drinking was collected only once per day within a consistent two-hour window, preventing us from establishing the within-day temporal sequence of PTSD symptoms, affect, and drinking behavior. A strength of the present study is its goal of establishing the lag time in which correlations between PTSD symptom features and drinking outcomes were strongest rather than pre-supposing the temporal aspect of such associations as concurrent or immediately subsequent. The present method does, however, assume that symptoms experienced over a 24-h period affect subsequent 24-h periods, when within-day fluctuations or the time of day in which symptoms are experienced may have unique effects on drinking behavior. Fortunately, future research that overcomes this limitation is feasible. Prior micro-longitudinal studies have successfully collected data on PTSD and drinking behavior from four (Possemato et al., 2012) to eight (Gaher et al., 2014) times daily, and a review of this literature did not find that repeated micro-longitudinal monitoring, itself, increased or decreased alcohol use or PTSD symptoms (Lane et al., 2019). Scholarship on PTSD and alcohol use may greatly benefit from applying the present analytic approach to a larger sample with multiple, random same-day assessment points. The retrospective nature of recalling PTSD and drinking concurrently may have influenced participants’ responses. The study also did not assess daily drinking-related consequences. Although some negative consequences of drinking occur infrequently and are less likely to be a factor in micro-longitudinal studies, in cross-sectional and macro-longitudinal studies consequences are likely to show associations with negative affect and PTSD symptoms (Read et al., 2012; Tripp et al., 2015). Another limitation lies in the sample composition, which consists of women attending a competitive college and potentially excludes those with greater difficulty related to trauma history or drinking who might be unable to attend school. The study’s focus on women with a history of SV and a PTSD diagnosis who engage in higher risk drinking further limits generalizability to a broader population and limits the conclusions that can be drawn regarding the stability of PTSD symptoms to a higher drinking sample. These findings would need to be replicated within a broader sample of individuals with PTSD, regardless of drinking levels. The relatively small size of our sample limited our ability to account for demographic covariates and to investigate whether there were differential effects of childhood abuse versus sexual victimization during adolescence or adulthood (Walker et al., 2021; Boumpa et al., 2022). It is possible this sample was biased by the effects of PTSD among some women in our target population. PTSD symptoms such as avoidance of reminders and difficulty concentrating, may have been barriers to participation among women who were experiencing these symptoms more strongly. In addition, those with the most severe PTSD may drop out of college and never have the opportunity to be included in the research from college settings. Finally, data used in these secondary analyses were collected shortly before 2013, utilizing subjective mood assessments and measures of PTSD aligned with DSM-IV criteria. This limits the interpretation of findings in the context of the current DSM-5 criteria. Although there is generally good agreement between DSM-IV and DSM-5 findings (Rosellini et al., 2015; Crespo and Gómez, 2016), future research should aim to validate these findings using current assessments.

In conclusion, this study provides valuable insights into the short-term, longitudinal stability and predictive dynamics of PTSD symptoms, affect, and drinking behavior among college women exposed to SV. Our findings highlight the chronic and severe nature of PTSD symptoms and their significant impact on emotional regulation and alcohol cravings. The observed stability in PTSD symptoms and the variability in affect suggest the importance of targeting both persistent symptoms and emotional dysregulation in interventions. The identification of time-lagged associations between specific PTSD symptoms and drinking behavior, with delays of 3–4 days, contrasts with the immediate effects assumed in the self-medication hypothesis (i.e., same or next day). This underscores the complexity of these interactions and the need for comprehensive therapeutic approaches that consider longer-range effects. Additionally, our choice of cohort, which included individuals who already engage in binge drinking, suggests that cravings are a critical variable to assess in populations where drinking behavior is normalized due to social factors. Future research should expand on these exploratory findings by incorporating longer-range temporal assessments and exploring a broader range of symptom interactions. Addressing the psychological and environmental factors influencing alcohol use in individuals with PTSD can lead to more effective and tailored intervention strategies.

## Data availability statement

The data that support the findings of this study are available from the corresponding author upon reasonable request.

## Ethics statement

The studies involving humans were approved by University of Washington Institutional Review Board. The studies were conducted in accordance with the local legislation and institutional requirements. The participants provided their written informed consent to participate in this study.

## Author contributions

SB: Conceptualization, Formal analysis, Funding acquisition, Investigation, Methodology, Visualization, Writing – original draft, Writing – review & editing. MS: Conceptualization, Formal analysis, Writing – review & editing. TW: Conceptualization, Funding acquisition, Writing – original draft. MK: Resources, Supervision, Writing – review & editing. DK: Conceptualization, Data curation, Funding acquisition, Supervision, Writing – review & editing.
