# Supplementary material for: A month in review: longitudinal dynamics between daily PTSD symptom networks, affect, and drinking behaviors in female college students
Source: Front Psychol. 2024 Jul 30;15:1388539. doi: 10.3389/fpsyg.2024.1388539 (PMC11319128; doi:10.3389/fpsyg.2024.1388539)
Supplement: Supplementary file 1 [file Table_1.DOCX]

Supplementary Material


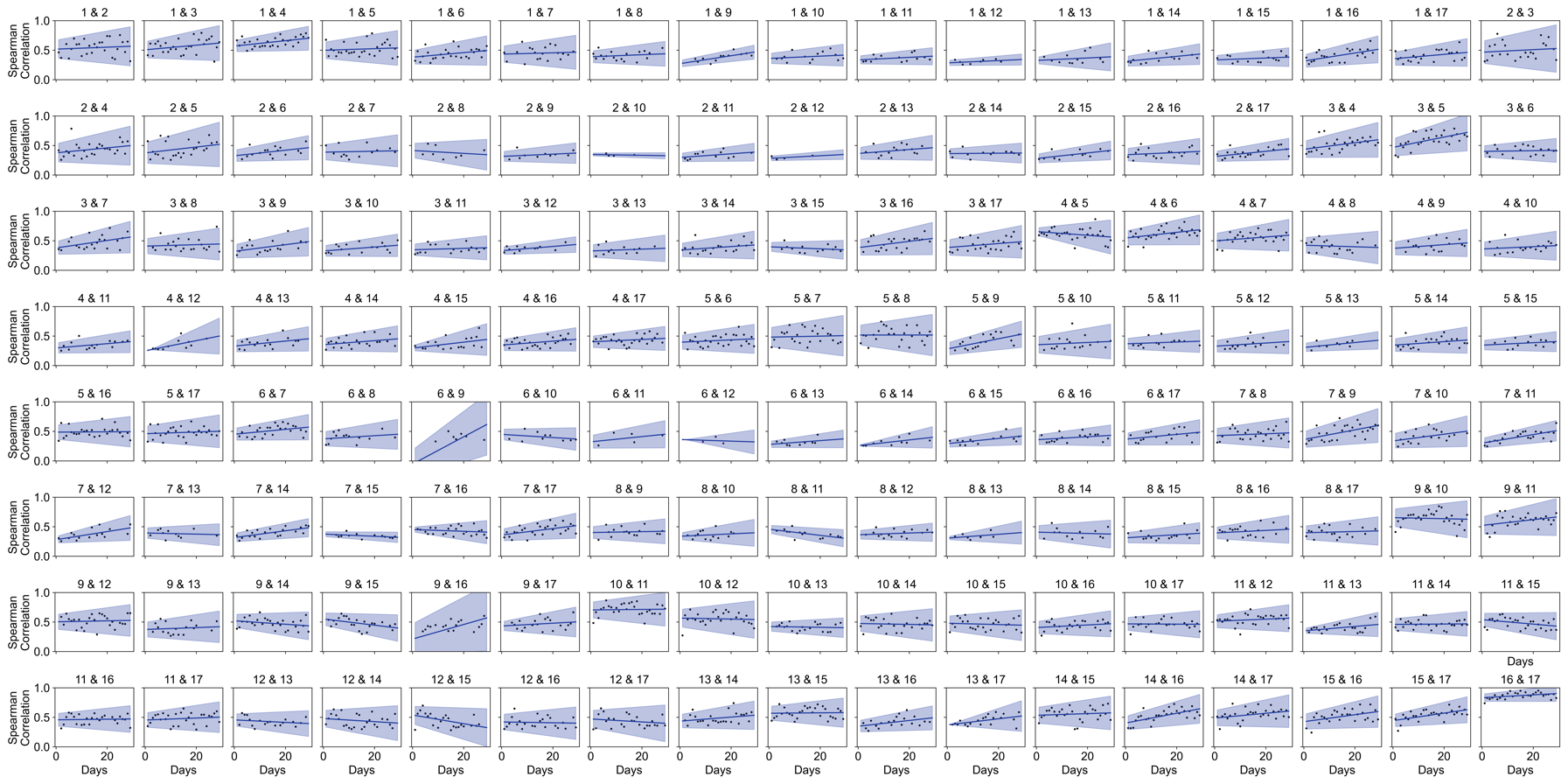


**Supplementary Figure 1. Fitted models for individuals with PTSD.** The fitted model for each edge weight in the symptom network can be described by four metrics: (1) the temporal expectation of the model mean *[µ]* (i.e., the average predicted correlation value at day 15), (2) the temporal change of the model mean  *[Δµ]* (i.e., the predicted correlation slope that indicates how much the mean changes over time), (3) the temporal expectation of the model’s standard deviation [*σ*] (i.e., the average predicted standard deviation at day 15), and (4) the temporal change of the model’s standard deviation *[Δσ]* (i.e., the predicted standard deviation slope that indicates how much the standard deviation changes over time). Each panel illustrates the temporal evolution of symptom correlations. The central blue line denotes the mean prediction from the linear Gaussian model, with the surrounding shaded area representing the 95% confidence interval. Individual daily symptom correlation values are depicted as black dots. The symptoms include: (1) intrusive memories, (2) nightmares, (3) flashbacks, (4) distress at reminders, (5) physiological arousal at reminders, (6) avoidance of thoughts/feelings, (7) avoidance of activities and situations, (8) psychogenic amnesi, (9) loss of interest, (10) emotional isolation, (11) emotional numbing, (12) foreshortened future, (13) sleep disturbance, (14) irritability, (15) concentration problems, (16) hypervigilance, and (17) excessive startle.


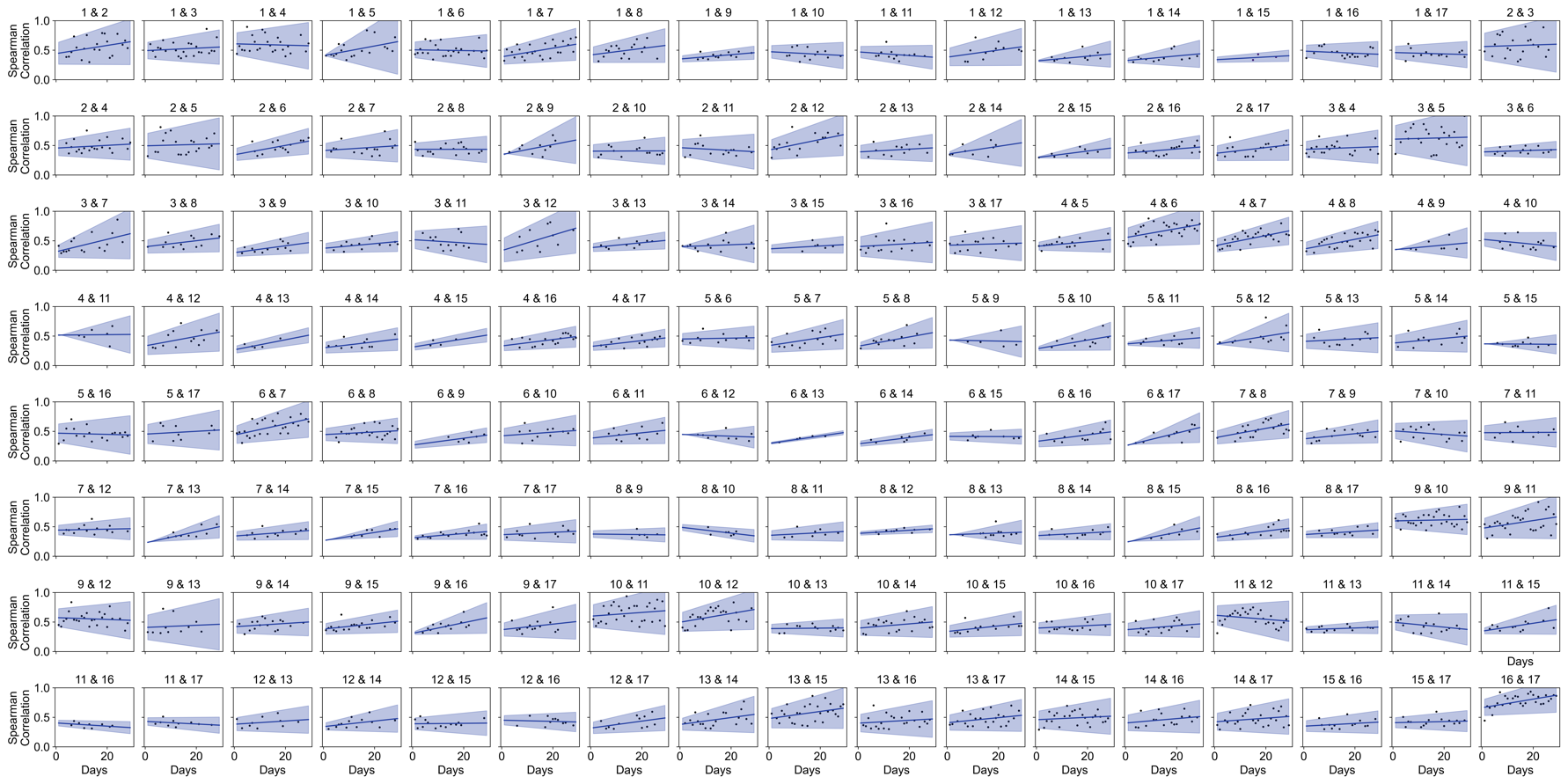


**Supplementary Figure 2. Fitted models for the SV-exposed group.** The fitted model for each edge weight in the symptom network can be described by four metrics: (1) the temporal expectation of the model mean *[µ]* (i.e., the average predicted correlation value at day 15), (2) the temporal change of the model mean  *[Δµ]* (i.e., the predicted correlation slope that indicates how much the mean changes over time), (3) the temporal expectation of the model’s standard deviation [*σ*] (i.e., the average predicted standard deviation at day 15), and (4) the temporal change of the model’s standard deviation *[Δσ]* (i.e., the predicted standard deviation slope that indicates how much the standard deviation changes over time). Each panel illustrates the temporal evolution of symptom correlations. The central blue line denotes the mean prediction from the linear Gaussian model, with the surrounding shaded area representing the 95% confidence interval. Individual daily symptom correlation values are depicted as black dots. The symptoms include: (1) intrusive memories, (2) nightmares, (3) flashbacks, (4) distress at reminders, (5) physiological arousal at reminders, (6) avoidance of thoughts/feelings, (7) avoidance of activities and situations, (8) psychogenic amnesi, (9) loss of interest, (10) emotional isolation, (11) emotional numbing, (12) foreshortened future, (13) sleep disturbance, (14) irritability, (15) concentration problems, (16) hypervigilance, and (17) excessive startle.


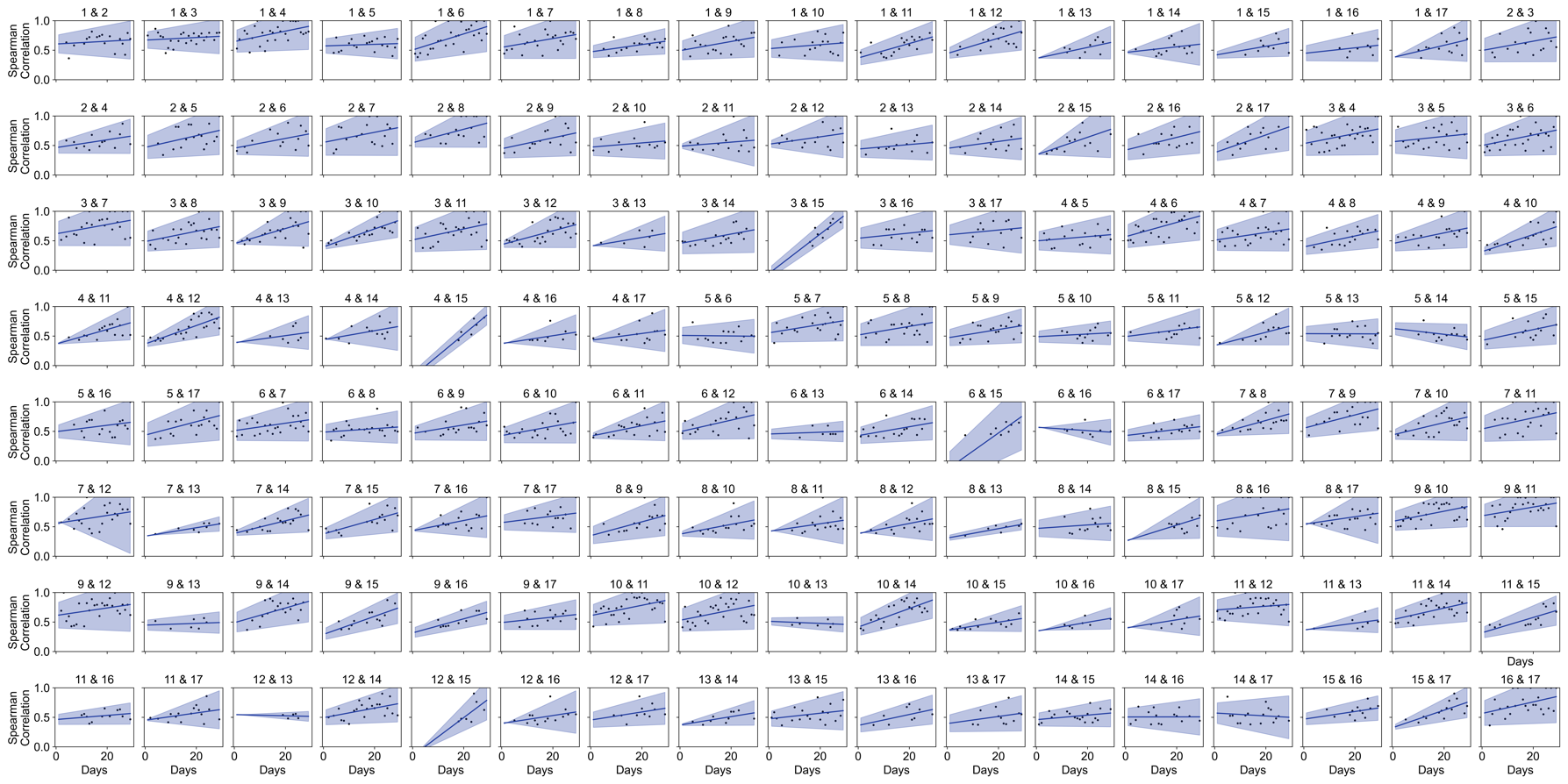


**Supplementary Figure 3. Fitted models for the no-trauma group.** The fitted model for each edge weight in the symptom network can be described by four metrics: (1) the temporal expectation of the model mean *[µ]* (i.e., the average predicted correlation value at day 15), (2) the temporal change of the model mean  *[Δµ]* (i.e., the predicted correlation slope that indicates how much the mean changes over time), (3) the temporal expectation of the model’s standard deviation [*σ*] (i.e., the average predicted standard deviation at day 15), and (4) the temporal change of the model’s standard deviation *[Δσ]* (i.e., the predicted standard deviation slope that indicates how much the standard deviation changes over time). Each panel illustrates the temporal evolution of symptom correlations. The central blue line denotes the mean prediction from the linear Gaussian model, with the surrounding shaded area representing the 95% confidence interval. Individual daily symptom correlation values are depicted as black dots.The symptoms include: (1) intrusive memories, (2) nightmares, (3) flashbacks, (4) distress at reminders, (5) physiological arousal at reminders, (6) avoidance of thoughts/feelings, (7) avoidance of activities and situations, (8) psychogenic amnesi, (9) loss of interest, (10) emotional isolation, (11) emotional numbing, (12) foreshortened future, (13) sleep disturbance, (14) irritability, (15) concentration problems, (16) hypervigilance, and (17) excessive startle.

**B**

**A**


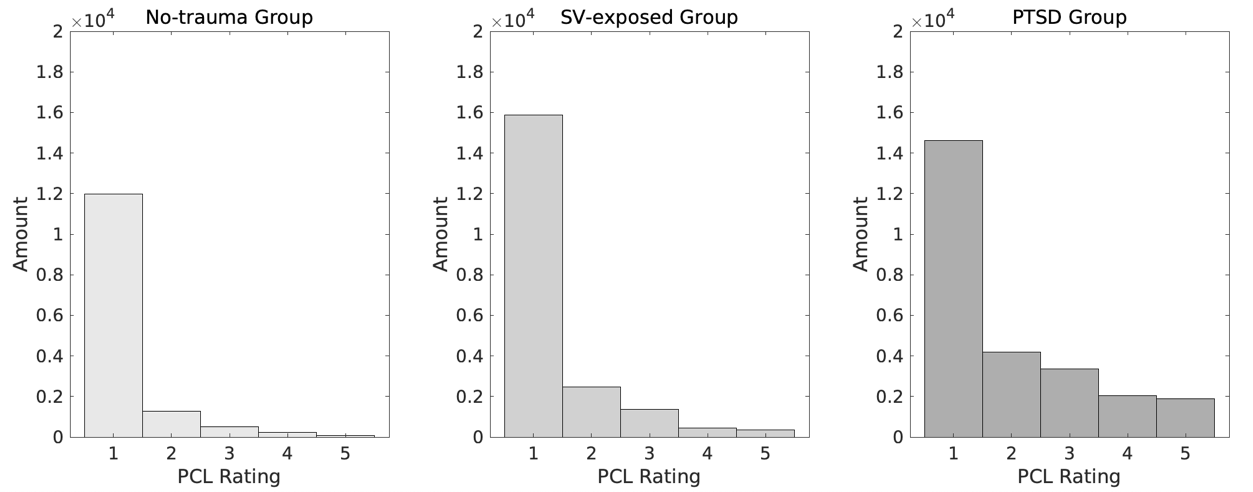


**Figure S4. Group differences between total PTSD symptom severity.** To ensure the validity of our analyses, we first calculated the mean total PTSD symptom severity (i.e., the summed score across all 17 PTSD symptoms) for each participant over the four weeks. We then compared these mean scores between the groups using a Kruskal-Wallis test to confirm that the PTSD group exhibited greater symptom severity. Post-hoc multiple comparisons were FDR-corrected. As shown in **A**, the validation analysis confirmed significant differences in total PTSD symptom severity between the groups (χ²(2) = 50.019, *p* < .001). The PTSD group exhibited the highest PTSD symptom severity (adjusted *p* < .001), followed by the SV-exposed group, which had higher severity than the no-trauma group (adjusted *p* = .02). Statistically significant differences are denoted with *** at FDR-corrected p < .001 and * at p < .05. (**B**) We also provide the corresponding histograms that display raw PCL ratings for each group across all participants and days, offering an overview of the within-group distribution.

**Figure S5. Mean scores and standard deviations across the affect and drinking behavior measures.** We calculate the *mean scores* over the 29 days to provide a baseline assessment of the central tendency of the data. We also assessed the *standard deviations* over the 29 days, indicated by the bars in the plot, to provide insights into the variability or spread of your data across days (i.e., a consistent standard deviation across days suggests stability in the dispersion of your data, while significant changes may indicate fluctuations in variability).

PTSD Group

No-trauma Group

SV-exposed Group

**Figure S6. Absolute day-to-day differences in mean scores and standard deviations.** We calculated the *standard deviations* over the 29 days to provide insights into the variability or spread of your data across days (i.e., a consistent standard deviation across days suggests stability in the dispersion of your data, while significant changes may indicate fluctuations in variability). Additionally, we derived the *absolute (day-to-day) standard deviation differences* to assess how much the standard deviation varies from one day to the next without considering the direction of change (i.e., a smaller absolute difference implies greater stability in the variability of your measure over the observed period.

PTSD Group

SV-exposed Group

No-trauma Group
